# Staged Tendon Repair to Improve Range of Motion in Tamai Zone 4 Replantation: Two Case Reports

**DOI:** 10.1055/a-2190-8487

**Published:** 2024-02-28

**Authors:** Takeo Matsusue

**Affiliations:** 1 Department of Plastic Reconstructive Surgery, Kansai Electric Power Hospital, Osaka, Japan

**Keywords:** replantation, range of motion, articular, rehabilitation, tendon, finger

## Abstract

Tamai zone 4 replantation, defined as the replantation at a level proximal to the flexor digitorum superficialis' insertion and distal to where the common digital artery branches into the proper digital artery, has poor functional results because making orthosis and rehabilitation protocols that protect the bone and the flexor and extensor tendons simultaneously difficult. Two cases of Tamai zone 4 replantation are presented: one case of an index finger replantation at the proximal phalanx and a case of ring finger replantation at the proximal interphalangeal joint. The author did not repair the flexor tendon intentionally in the primary replantation and performed two-stage flexor tendon reconstruction later. The total active motions (TAMs) at the last follow-up were 215 and 180 degrees, respectively, with the latter distal interphalangeal joint being an arthrodesis. Both cases had no extension lag in the proximal interphalangeal joint. These results were much better than those in previous reports, in which the mean TAM was 133 degrees or less. The good results appeared to be mainly due to the reasonable and clear postoperative rehabilitation protocols made by the proposed procedure. This procedure may be useful for obtaining reproducible functional results even in Tamai zone 4 replantation.

## Introduction

Treatment methods and postoperative therapy for finger tendon isolated injury have been established, and good results have been obtained. 1 However, the functional results are poor in replanted fingers, especially in the replantation at a level proximal to the flexor digitorum superficialis' insertion and distal to where the common digital artery branches into the proper digital artery (Tamai zone 4 replantation) in which all structures are damaged simultaneously and need repair. 2 3 A single-digit Tamai zone 4 replantation is regarded as relatively contraindicated because it is functionally inferior to amputation stump plasty. 4 Nevertheless, if a surgeon performs Tamai zone 4 replantation, it is necessary to keep in mind that survival alone is not enough and that excellent functional outcomes with joint mobility are required to overrule rationales for that relative contraindication.

Traditionally, the flexor and extensor tendons are repaired simultaneously during the replantation. 5 Early motion exercises would be necessary for obtaining good functional results with Tamai zone 4 replantation. However, it is difficult to make an orthosis that does not affect blood circulation immediately after replantation and also protects the bone and the flexor and extensor tendons simultaneously. 3 Unfortunately, no clear reproducible method and postoperative rehabilitation protocol can produce good functional results of replanted fingers in Tamai zone 4. 6 Surgeons should treat injuries to multiple anatomic structures based on previous findings on isolated injuries to the various component structures. 3

Some reports have indicated the effectiveness of staged flexor reconstruction for severely scarred digits. 7 The standard postoperative rehabilitation protocol for flexor tendon repair is early motion therapy; whereas for extensor tendon repair and bone fracture fixation, it is immobilization. 7 8 Therefore, rehabilitation after extensor tendon repair is more consistent with treatment after bone fracture fixation than that of flexor tendon repair. Based on these findings, the author hypothesized that, if the flexor tendons were not intentionally repaired during primary replantation, with only the extensor tendons repaired, and then the flexor tendons were reconstructed in two stages, rehabilitation would be simple and clear, and good functional results with high reproducibility would be obtained. Under this hypothesis, the author performed two cases of Tamai zone 4 replantation. This report aimed to describe the details and functional results of this method and to discuss its effectiveness.

## Case

### Case 1



**Video 1** At 7 years and 9 months after the replantation for a crush amputation of the index finger at the proximal phalanx.


**Fig. 1:**
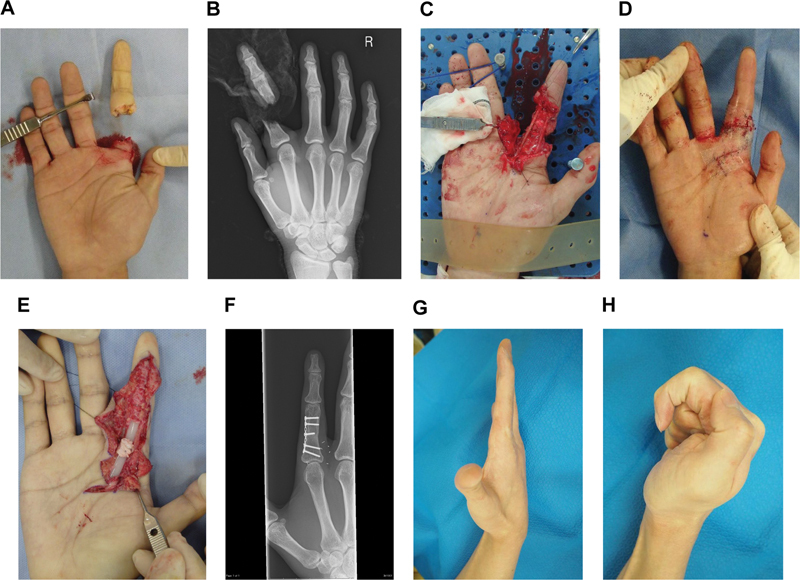
Case 1: ( **A** ) A crush amputation of the index finger at the proximal phalanx. ( **B** ) X-ray image at the time of injury. ( **C** ) Intraoperative view of the replantation. ( **D** ) Immediately after the replantation, the proximal interphalangeal joint was kept at an extended position without any orthosis. ( **E** ) Intraoperative view of the second surgery: a silicone rod insertion and the A2 pulley reconstruction. ( **F** ) X-ray image at 18 months after the replantation. ( **G** ) At 7 years and 9 months after the replantation, the patient achieved full extension of the proximal interphalangeal joint. ( **H** ) At 7 years and 9 months after the replantation, the total active motion was 215 degrees.

A 20-year-old healthy man sustained a crush amputation of the right index finger at the proximal phalanx while he was working ( Fig. 1A, B ). He provided written informed consent, and the replantation under axillary nerve block anesthesia was begun 5 hours postinjury. The dissection around the extensor tendons was kept to a minimum to minimize postoperative adhesion. Bone, extensor tendon, bilateral digital nerve, ulnar digital artery, and two dorsal veins were repaired. The author did not repair flexor tendons during this replantation ( Table 1 ). Because of the risk of infection of the silicone rod tendon spacer, which could spread beyond the local area to the forearm, the silicone rod was not inserted during primary replantation ( Fig. 1C ). Loose bulky dressing was applied, and a simple volar brace was attached in the wrist extension position. The operation took 6 hours and 20 minutes ( Fig. 1D ). On day 8 after the replantation, the dorsal blocking plastic splint was applied to protect the proximal phalanx and extensor tendon with 45 degrees of extension in the wrist, 50 degrees of flexion in the metacarpophalangeal joints (MCPJs), and 0 degree in the proximal interphalangeal joint (PIPJ) and distal interphalangeal joint (DIPJ). Exercise on the unaffected parts of the hand was started while protecting the fractured part. From 4 weeks after the replantation, the static splint was changed to the index finger only, and the fractured part and extensor tendon were continued to be protected. Bone fusion was confirmed at 9 weeks after replantation. Accordingly, the static splint was removed, and passive exercise for the index finger was started. The extension contracture was released after approximately 4 months of exercise, mainly using dynamic splints. No surgical procedure was performed to release the extension contracture. At 5 months after the replantation, the second surgery was performed. A silicone rod was inserted. The A3, A4, and A5 pulleys were preserved. The A1 and A2 pulleys were defective, and the A2 pulley was reconstructed to limit the risk for bowstringing ( Table 1 ; Fig. 1E ). Due to the risk of poor wound healing in the scar tissue area, rehabilitation was suspended for 2 weeks after the second surgery, to prioritize wound healing. After 3 more months, the third surgery was performed. The silicone rod was removed, and the right palmaris longus (PL) tendon was grafted ( Table 1 ). The dorsal blocking plastic splint was applied with 20 degrees of extension in the wrist, 60 degrees of flexion in the MCPJ, and 0 degree in the PIPJ and DIPJ. On the next day, the combined regimen of modified Kleinert and modified Duran techniques was started. 9 Three weeks after tendon grafting, active flexion exercise was started while applying the dorsal blocking plastic splint. Six weeks after tendon grafting, the orthosis was removed, and passive extension exercise was started. At 12 weeks after tendon grafting, muscle strengthening exercise was started. At 4 months after tendon grafting, the exercise regimes were finished. Thereafter, only follow-up observations were made. At the last follow-up (7 years and 9 months after the replantation), the total active motion (TAM) was 215 degrees ( Table 2 ); the Semmes–Weinstein monofilament test (SWT) results were 2.83 and 3.61 in radial side and ulnar side, respectively; static two-point discrimination (s2PD) was 10 mm; and the moving two-point discrimination (m2PD) was 9 mm ( Table 3 ). 10 11 The finger length and contour were preserved ( Fig. 1F–H ; Video 1 , available in the online version only). The Disabilities of Arm, Shoulder, and Hand (DASH) score was 1.72.

**Table 1:** Injury conditions and main procedures in case 1

Tissue	Primary condition	Replantation	Second surgery	Third surgery
**Proximal phalanx**	Transverse fracture	Not shortened Fixed by screws and a plate	–	–
**Bilateral lateral bands**	Disrupted at the same level as the fracture	Repaired by 5–0 nylon horizontal mattress suture	–	–
**Central slip**	Disrupted at the same level as the fracture	Repaired by 5–0 nylon horizontal mattress suture and 6–0 nylon cross-stitch suture	–	–
**Flexor tendons**	Disrupted at the same level as the fracture	Not repaired intentionally Flexor tendons were left as spacers in A3, A4, and A5 pulleys	Flexor tendon remnants in finger and palm were excised Silicone rod insertion c A2 pulley reconstruction using the excised FDP tendon	Silicone rod was removed, and PL tendon was grafted. The distal graft end was inserted to the distal phalanx with 4–0 nylon pullout suture, and the proximal end was stitched to the FDS tendon of the index finger with 4–0 nylon interweave suture at the wrist d
**Radial digital nerve**	15-mm long defect a	Reconstructed using a nerve conduit b	–	–
**Ulnar digital nerve**	30-mm long defect a	Reconstructed using a nerve conduit b	–	–
**Ulnar digital artery**	20-mm long defect a	Reconstructed using a vein graft from the distal forearm	–	–
**Veins**	Disrupted at the same level as the fracture	Two dorsal veins were anastomosed directly	–	–

Abbreviations: FDP, flexor digitorum profundus; FDS, flexor digitorum superficialis; PL, palmaris longus.

a After debridement.

b Polyglycolic acid collagen nerve conduit filled with collagen sponge (Nerbridge; Toyobo, Osaka, Japan). The diameter was 1.5 mm.

c A silicone rod was passed through the pulley system. The distal end of the silicone rod was sutured distally to the distal FDP tendon stump. The proximal end was left free in the forearm.

d The FDS was selected to facilitate individual movement of the index finger. The positions of the joints at graft finalization were adjusted to match the resting joint positions of the adjacent finger.

**Table 2:** Range of motion in case 1

Period after flexor tendon reconstruction	MCPJ AROM (E/F)	MCPJ PROM (E/F)	PIPJ AROM (E/F)	PIPJ PROM (E/F)	DIPJ AROM (E/F)	DIPJ PROM (E/F)	TAM	Percent TAM a
Immediately before	0/90	20/90	0/25	0/95	−10/20	0/80	125	46.3%
4 months	0/90	20/90	0/75	0/90	−25/50	0/75	190	70.4%
16 months	0/90	30/90	0/82	0/90	−20/55	0/60	207	76.7%
85 months	0/95	45/100	0/85	0/90	−25/60	−5/80	215	79.6%

Abbreviations: AROM, active range of motion; DIPJ, distal interphalangeal joint; E/F, extension/flexion; MCPJ, metacarpophalangeal joint; PIPJ, proximal interphalangeal joint; PROM, passive range of motion; TAM, total active motion.

Values are presented as degrees.

a Calculated by dividing the TAM of the injured finger by the TAM of the contralateral healthy finger.

**Table 3:** Sensory status in case 1

	SWT		s2PD	m2PD
	Radial side	Ulnar side		
Right index finger				
24 months after replantation	3.22	4.17	One point a	One point a
93 months after replantation	2.83	3.61	10	9
Left index finger	2.83	2.83	4	3

Abbreviations: m2PD, moving two-point discrimination; SWT, Semmes–Weinstein monofilament test; s2PD, static two-point discrimination.

a Failure to distinguish two points.

### Case 2



**Video 2** At 20 months after the replantation for an avulsion amputation of the ring finger at the proximal interphalangeal joint.


**Fig. 2:**
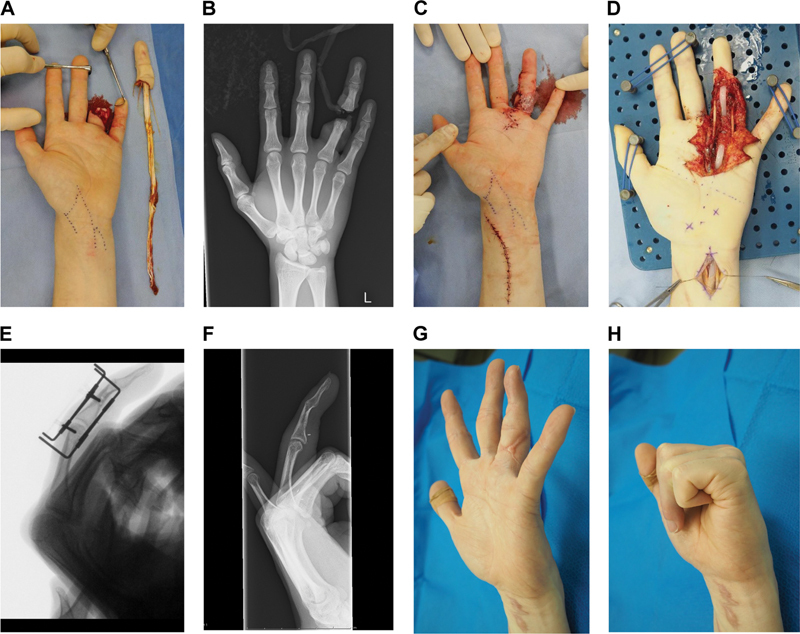
Case 2: ( **A** ) An avulsion amputation of the ring finger at the proximal interphalangeal joint. ( **B** ) X-ray image at the time of injury. ( **C** ) Immediately after the replantation, the proximal interphalangeal joint was kept at the extended position without any orthosis. ( **D** ) Intraoperative view of the second surgery: a silicone rod insertion, bilateral nerve reconstructions, and the distal interphalangeal joint arthrodesis. ( **E** ) X-ray image immediately after the third surgery: the tendon grafting and the proximal interphalangeal joint skeletal traction. ( **F** ) X-ray image at 20 months after the replantation. ( **G** ) At 20 months after the replantation, the patient achieved full extension of the proximal interphalangeal joint. ( **H** ) At 20 months after the replantation, the total active motion was 180 degrees.

A 23-year-old healthy man sustained an avulsion amputation of the left ring finger at the PIPJ while he was working ( Fig. 2A, B ). He provided written informed consent, and the replantation under axillary nerve block anesthesia was begun 4.5 hours postinjury. PIPJ, central slip, radial digital artery, and two dorsal veins were repaired. The author did not repair the terminal extensor, flexor tendons, and bilateral digital nerves during this replantation ( Table 4 ). Loose bulky dressing was applied, and a simple volar brace was attached in the wrist extension position. The operation took 6.5 hours ( Fig. 2C ). On day 8 after the replantation, the volar blocking plastic splint was applied to protect the PIPJ and extensor tendon with 30 degrees of extension in the wrist, 0 degree in the MCPJ, PIPJ, and DIPJ, and started exercise on the unaffected parts of the hand. From 4 weeks after the replantation, passive exercise for the ring finger was started. The extension contracture was released after approximately 4 months of exercise, mainly using dynamic splints. No surgical procedure was performed to release the extension contracture. Five months after the replantation, the second surgery was performed. A silicone rod was inserted, and bilateral nerve reconstructions and DIPJ arthrodesis were performed simultaneously. The A1, A2, and A4 pulleys were preserved ( Fig. 2D ; Table 4 ). Similar to case 1, rehabilitation was suspended for 2 weeks after the second surgery. After the next 3 months, the third surgery was performed. The silicone rod was removed, and the left PL tendon was grafted. In addition, the PIPJ was in skeletal traction for 4 weeks by DDA-2 external fixator (ME System, Tokyo, Japan) because the author was concerned that a sudden load on the PIPJ could cause osteoarthritis ( Fig. 2E ; Table 4 ). The DDA-2 external fixator maintains the mobility of the PIPJ and traction on the joint is provided by the rubber band. The range-of-motion exercises were started with the external fixator applied. The rehabilitation protocol and duration after flexor tendon grafting were the same as in case 1. At the last follow-up (20 months after the replantation), the TAM was 180 degrees ( Table 5 ). The SWT results were 3.84 and 4.56 in radial and ulnar sides, respectively, the s2PD was 12 mm, and the m2PD was not able to distinguish two points ( Table 6 ). The finger length and contour were preserved ( Fig. 2F–H ; Video 2 , available in the online version only). The DASH score was 0.83.

**Table 4:** Injury conditions and main procedures in case 2

Tissue	Primary condition	Replantation	Second surgery	Third surgery
**PIPJ**				Skeletal traction by DDA-2 external fixator
** Radial collateral ligament**	Avulsed at the proximal phalanx with small bone fragment	Sutured to the proximal phalanx with 4–0 nylon	–	–
** Ulnar collateral ligament**	Avulsed at their insertions in the middle phalanx	Sutured to the middle phalanx with 4–0 nylon	–	–
** Volar plate**	Avulsed at their insertions in the middle phalanx	Sutured to the middle phalanx with 4–0 nylon	–	–
**Central slip**	Avulsed at their insertions in the middle phalanx	Sutured to the middle phalanx with 4–0 nylon	–	–
**Terminal extensor**	Severely damaged and avulsed at its insertion in the distal phalanx	Not repaired because obtaining active extension of the DIPJ would be difficult, even if it was repaired	DIPJ arthrodesis	–
**Flexor tendons**	Avulsed at the musculotendinous junction	Not repaired intentionally Flexor tendons were excised proximal to the A1 pulley and inserted into the pulleys as spacers	Tendon remnants were excised Silicone rod insertion b A1, A2, and A4 pulleys were preserved	Silicone rod was removed. PL tendon was grafted. The distal graft end was anchored to the middle phalanx, and the proximal end was stitched to the FDS tendon of the middle finger with 4–0 nylon interweave suture at the wrist d
**Radial digital nerve**	Severely damaged and avulsed from the base of the finger	Not repaired because it was difficult to determine the extent of debridement	30-mm long defect a was reconstructed using a nerve conduit c	–
**Ulnar digital nerve**	Severely damaged and avulsed from the base of the finger	Not repaired because it was difficult to determine the extent of debridement	20 mm long defect a was reconstructed using a nerve conduit c	–
**Radial digital artery**	30-mm long defect a	Reconstructed using a vein graft from the distal forearm		
**Veins**	Disrupted at PIPJ	Two dorsal veins were anastomosed directly		

Abbreviations: DIPJ, distal interphalangeal joint; FDS, flexor digitorum superficialis; PIPJ, proximal interphalangeal joint; PL, palmaris longus.

a After debridement.

b A silicone rod was passed through the pulley system and sutured distally to the distal FDP tendon stump. The proximal end of the silicone rod was left free in the forearm.

c Polyglycolic acid collagen nerve conduit filled with collagen sponge (Nerbridge; Toyobo, Osaka, Japan). The diameter was 1.5 mm.

d The FDS was selected because of the risk of quadriga syndrome. The positions of the joints at graft finalization were adjusted to match the resting joint positions of the adjacent finger.

**Table 5:** Range of motion in case 2

Period after flexor tendon reconstruction	MCPJ AROM (E/F)	MCPJ PROM (E/F)	PIPJ AROM (E/F)	PIPJ PROM (E/F)	DIPJ AROM (E/F)	DIPJ PROM (E/F)	TAM	Percent TAM a
**Immediately before**	0/85	0/85	0/0	0/95	−10/10 b	−10/10 b	85	31.5%
**5 months**	0/85	0/85	0/90	0/90	−10/10 b	−10/10 b	175	64.8%
**12 months**	0/90	30/90	0/90	0/90	−10/10 b	−10/10 b	180	66.7%

Abbreviations: AROM, active range of motion; DIPJ, distal interphalangeal joint; E/F, extension/flexion; MCPJ, metacarpophalangeal joint; PIPJ, proximal interphalangeal joint; PROM, passive range of motion; TAM, total active motion.

Values are presented as degrees.

a Calculated by dividing the TAM of the injured finger by the TAM of the contralateral healthy finger.

b Distal interphalangeal joint arthrodesis.

**Table 6:** Sensory status in case 2

	SWT		s2PD (mm)	m2PD (mm)
	Radial side	Ulnar side		
Left ring finger				
20 months after replantation	3.84	4.56	12	One point a
Right index finger	2.83	2.83	4	3

Abbreviations: m2PD, moving two-point discrimination; SWT, Semmes–Weinstein monofilament test; s2PD, static two-point discrimination.

a Failure to distinguish two points.

## Discussion

In the two cases of Tamai zone 4 replantation presented, the tendon repair in the primary replantation was limited to the extensor tendon, and the flexor tendon was not intentionally repaired. Thereafter, the flexor tendon was reconstructed by two-stage flexor tendon reconstruction. As a result, the TAM at the last follow-up was 215 degrees in one case and 180 degrees with DIPJ arthrodesis in the other. Both cases had no extension lag in the PIPJ.

Most reports of Tamai zone 4 replantation described poor functional recovery. In reports of Tamai zone 4 replantation since 2000, Chen et al stated that the average TAM of five single-digit replantation cases was 97 degrees, Buntic et al reported 133 degrees for nine single-digit replantation cases excluding those with DIPJ or PIPJ arthrodesis, Ross et al reported 126 degrees for replantation cases including multiple finger replantation and 17 degrees average extensor lag at the PIPJ. 3 12 13 Neither of the backgrounds of the two cases, such as age or extent of structural damage, were special compared with the cases in those reports. However, both cases obtained better range of motion and less extensor lag at the PIPJ than cases in those reports.

The presented procedure helps keep the PIPJ in the extended position without any orthosis after replantation because the flexor tendon is not repaired. Therefore, it has no risk of flexion contracture. The early motion exercise after replantation may cause undesired results such as kink and spasm of anastomosed vessels and nonunion. 3 14 However, the proposed procedure does not raise those concerns because the replanted finger is immobilized in the extended position until bone fusion is obtained. If the immobilized period is not too long, the extension contracture of the PIPJ appears to be improved by subsequent passive motion exercises without secondary surgical procedures.

When the flexor and extensor tendons are repaired, both tendons need to be protected postoperatively. However, no orthosis or universally accepted early mobilization protocol can protect both flexor and extensor tendons simultaneously. 6 The force of the flexor tendon is stronger than that of the extensor tendon after replantation, which causes disruption or elongation of the repaired extensor tendon. 3 15 The author's postoperative program for extensor tendon was the traditional prolonged immobilization, followed by gradual mobilization in the absence of force from the flexor tendon. 8 The presented two cases had no extension lag in the PIPJ. If the repaired extensor tendon had sufficiently healed, the extensor tendon would not be disrupted or elongated even in the subsequent passive flexion exercise.

Silverman et al and Cheng et al reported cases of gaining the TAM of over 180 degrees in Tamai zone 4 replantation. 6 16 They both described that a skilled hand therapist is one of the basic requirements. 6 16 On the contrary, an outstandingly skilled hand therapist is not essential in the author's rehabilitation protocol. The reason is that the immobilization after extensor tendon repair and the two-stage flexor tendon reconstruction using a silicone rod are one of the standard treatments, and these rehabilitation protocols are written in many studies and have already been practiced in many institutions. 1 7 8 17

“Flexor tendon reconstruction during replantation followed by tenolysis” requires manipulation in severely scarred tissue. In contrast, the present method requires manipulation in severely scarred tissue during silicone rod insertion, but when performing the tendon graft, surgical manipulation is limited to the distal and proximal tendon sutures, and these sites are outside the zone of amputation injury. Therefore, there is less risk of postoperative adhesions. Basically, the primary repair of flexor tendons is generally contraindicated in cases of severe multiple tissue injuries to the finger. 18 The two-stage reconstruction has shown excellent results in restoring flexor tendon function to severely scarred digits. 7 The amputated finger is the most severe multiple tissue injury. Therefore, the author believes that it is rational to reconstruct the flexor tendon in two stages according to this principle. Pedicled sublimis tendon graft is another option for replacing the PL tendon graft with a two-stage reconstruction. 19 However, inserting a silicone rod at the primary replantation should be avoided due to the risk of infection.

Because replanted fingers may not survive, nerve reconstruction for nerve defects during replantation would be better performed with nerve conduits or allogeneic nerves than with relatively more invasive autologous nerve grafts. However, if staged tendon reconstruction is performed, nerve reconstruction should probably be done at the time of silicone rod insertion because during this, there is less risk of infection and it is easier to determine the extent of the nerve damage. The sensory deficiency of case 2 was worse than that of case 1, probably more due to the difference in the state of injury (crush in case 1, avulsion in case 2) than the timing of nerve reconstruction.

The extension lag at the DIPJ may be one of the limitations of the proposed procedure. In case 1, the patient had a 20 degrees extension lag at the DIPJ. Cheng et al also reported a 20 degrees extension lag at the DIPJ in their case report of excellent functional results. 16 Extensor tendon adhesions at the middle phalanx appear to be more difficult to improve with subsequent rehabilitation than those at the proximal phalanx.

Given the small number of cases, the findings should be interpreted with caution, and further studies using a larger cohort are needed to confirm the present findings. On the contrary, the proposed procedure could have indications to Tamai zone 5 or conditions other than replantation. For example, the toe-to-hand transfer and vascularized toe joint transfer, which reconstruct the bone, flexor tendon, and extensor tendon simultaneously, have the same problems, that is, postoperative rehabilitation is difficult and extension lag and joint contractures are likely to occur. 20

Herein, the author reported two cases of staged tendon repair for Tamai zone 4 replantation. These results were functionally much better than previous reports. The good results appeared to be mainly due to the reasonable and clear postoperative rehabilitation protocols made by the proposed procedure. The presented procedure may be useful for obtaining reproducible functional results even in Tamai zone 4 replantation.
